# Supplementary material for: The Plant Growth-Promoting Potential of Halotolerant Bacteria Is Not Phylogenetically Determined: Evidence from Two Bacillus megaterium Strains Isolated from Saline Soils Used to Grow Wheat
Source: Microorganisms. 2023 Jun 28;11(7):1687. doi: 10.3390/microorganisms11071687 (PMC10384442; doi:10.3390/microorganisms11071687)
Supplement: Supplementary file 1 [file microorganisms-11-01687-s001.zip › microorganisms-2455560-supplementary.pdf]

Supplementary data

Table S1 – Effect of increasing salinity and bacterial inoculation on wheat seedlings' water content (%). # indicates that for the control (no bacterial strain) under 250 mM of NaCl, all plants died. Values are the mean ± 1SE (n = 7 replicates).

[NaCl]	0 mM	150 mM	250 mM
Control	92 ± 1	96 ± 1	#
S1	92 ± 1	97 ± 0	98 ± 0
S2	93 ± 1	98 ± 1	97 ± 0
S3	92 ± 1	98 ± 0	98 ± 0
S4	93 ± 1	96 ± 1	97 ± 0

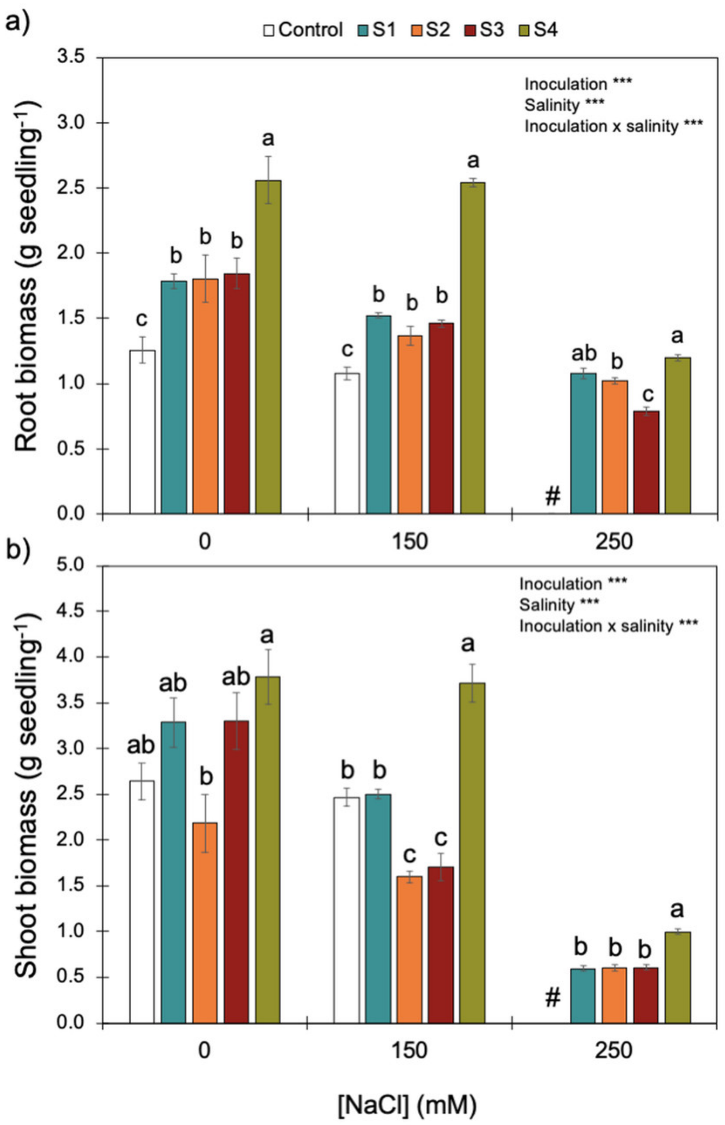

Figure S1 - Effect of increasing salinity and bacterial inoculation on wheat seedlings' root (**a**) and shoot biomass (**b**). *** shows significant effects ($p < 0.01$). Different letters show significant differences between bacterial strains for the same salinity level ($p < 0.05$). # indicates that for the control (no bacterial strain) under 250 mM of NaCl, all plants died. Bars are the mean ± 1SE (n = 7 replicates).

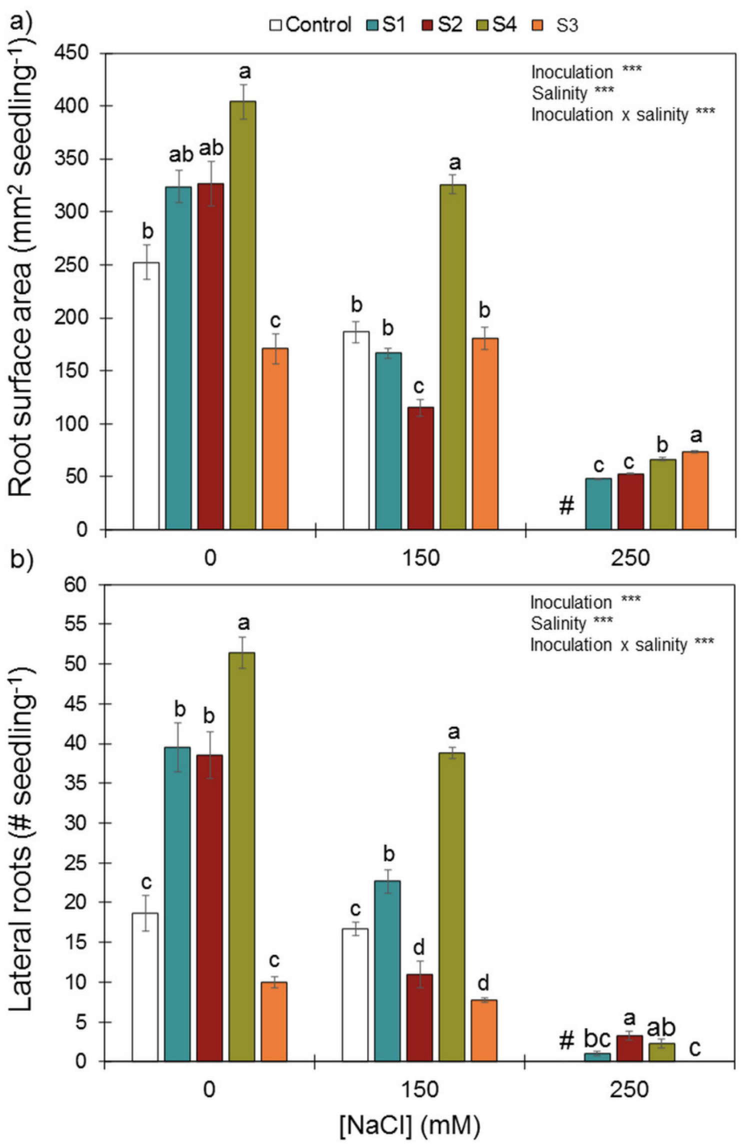

Figure S2 - Effect of increasing salinity and bacterial inoculation on the length of the wheat seedlings' root surface area (**a**), and the number of lateral roots (**b**). *** shows significant effects ($p < 0.01$). Different letters show significant differences between bacterial strains for the same salinity level ($p < 0.05$). # indicates that for the control (no bacterial strain) under 250 mM of NaCl, all plants died. Bars are the mean ± 1SE (n = 7 replicates).

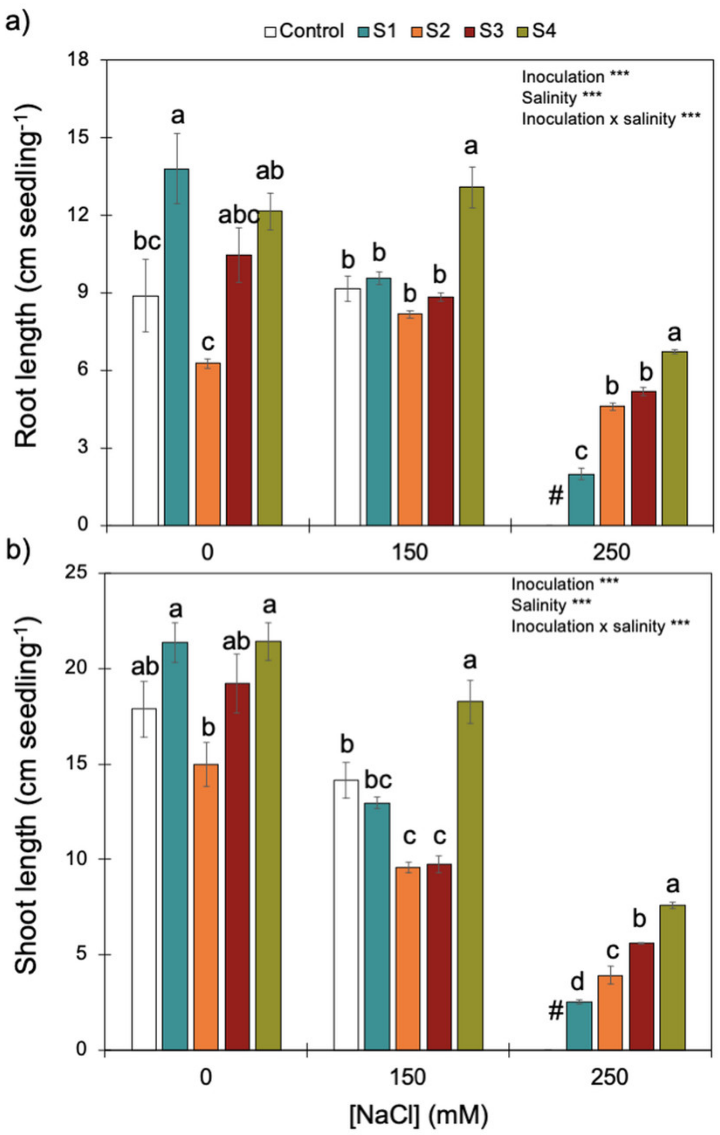

Figure S3 - Effect of increasing salinity and bacterial inoculation on the length of wheat seedlings' root (**a**), and shoot (**b**). *** shows significant effects ($p < 0.01$). Different letters show significant differences between bacterial strains for the same salinity level ($p < 0.05$). # indicates that for the control (no bacterial strain) under 250 mM of NaCl, all plants died. Bars are the mean ± 1SE (n = 7 replicates).
